# Diseases of *Cymbopogon citratus* (Poaceae) in China: *Curvularia nanningensis* sp. nov.

**DOI:** 10.3897/mycokeys.63.49264

**Published:** 2020-02-13

**Authors:** Qian Zhang, Zai-Fu Yang, Wei Cheng, Nalin N. Wijayawardene, Kevin D. Hyde4, Zhuo Chen, Yong Wang

**Affiliations:** 1 Department of Plant Pathology, Agriculture College, Guizhou University, Guiyang, Guizhou Province, 550025, China; 2 Department of Practaculture Science, Animal Science College, Guizhou University, Guiyang, Guizhou 550025, China; 3 Center for Yunnan Plateau Biological Resources Protection and Utilization, College of Biological Resource and Food Engineering, Qujing Normal University, Qujing, Yunnan 655011, China; 4 Center of Excellence in Fungal Research and School of Science, Mae Fah Luang University, Chiang Rai, 57100, Thailand; 5 Key Laboratory of Green Pesticide and Agricultural Bioengineering, Ministry of Education, Guizhou University, Guiyang 550025, China

**Keywords:** *
Cymbopogon
*, phylogeny, plant disease, Pleosporaceae, taxonomy

## Abstract

Five *Curvularia* strains isolated from diseased leaves of lemongrass (*Cymbopogon citratus*) in Guangxi Province, China, were examined. NCBI-Blast searches of ITS sequences suggested a high degree of similarity (99–100%) to *Curvularia akaii*, *C. akaiiensis*, *C. bothriochloae*, *C. heteropogonis* and *C. sichuanensis*. To accurately identify these strains, we further analysed their morphology and phylogenetic relationships based on combinations of ITS, GAPDH, and *tef*1 gene sequences. Morphological observations indicated that the key character differing from similar species was conidial size, whereas phylogenetic analyses indicated that the five strains represent one species that is also distinct from *C. akaii*, *C. akaiiensis* and *C. bothriochloae* by conidial size and conidiophore length. Thus, the strains examined are found to represent a new species described herein as *Curvularia nanningensis*. The pathogenicity test on the host and detached leaves confirmed the new species to be pathogenic on *Cymbopogon citratus* leaves. Standardised requirements for reliable identification of *Curvularia* pathogens are also proposed.

## Introduction

*Cymbopogon citratus* Stapf (lemongrass), believed to be a native of Malaysia, is now widely distributed in all continents and particularly in America, China, Guatemala and Southeast Asia. Essential oil from lemongrass is often used in aromatherapy (Williamson et al. 1996; Noel et al. 2002; Yang and Lei 2005; Shah et al. 2011). As a traditional Chinese medicine, lemongrass is known to provide relief from a variety of ailments including eczema, cold, headache and stomach-ache (Zhou et al. 2011). Guatemala is known to be the main exporter of lemongrass with about 250 tons per year. China produces 80 to 100 tons of lemongrass annually and the USA and Russia each imports about 70 tons per year (DAFF 2012). Depending on climatic conditions, lemongrass can be severely infected with a rust disease caused by *Puccinia nakanishikii* Dietel in Hawaii and California (Gardner 1985; Koike and Molinar 1999). In Brazil, a rust on lemongrass caused by another *Puccinia* species named *P. cymbopogonis* Massee has been reported (Vida et al. 2006). Joy et al. (2006) summarised the various disease symptoms and their causal agents of lemongrass.

*Curvularia* spp. infect many herbaceous plants including *Cymbopogon* Spreng. (Smith et al. 1989). *Helminthosporium cymbopogi* C.W. Dodge (≡ *Curvularia cymbopogonis* (C.W. Dodge) J.W.Groves & Skolko) is responsible for a severe disease of lemongrass in the lowlands of Guatemala (Dodge 1942). Barua and Bordoloi (1983) discovered *C. verruciformis* causing disease on *Cymbopogon flexuosus* Stapf. *Curvularia andropogonis* (Zimm.) Boedijn led to foliage blight of *Cymbopogon nardus* (L.) Rendle in the Philippines (Sato and Ohkubo 1990). Thakur (1994) reported *C. lunata* (Wakker) Boedijn as the causal agent of a new blight disease of Cymbopogon martini (Roxb.) Wats. var. motia Burk. Chutia et al. (2006) discovered that a leaf blight of *Cymbopogon winterianus* Jowitt is caused by *Curvularia* spp., resulting in a dramatic change in oil yield and its constituents. Recently, Santos et al. (2018) characterised the morphological and molecular diversity of the isolates of *C. lunata*, associated with *Andropogon* Linn. seeds.

Starting in 2010, there have been outbreak reports of pathogenic *Curvularia* in Asian countries, especially India and Pakistan (Pandey et al. 2014; Avasthi et al. 2015; Majeed et al. 2015). As China is a neighbouring country, we felt obligated to evaluate the potential threat of *Curvularia* to our crops. A severe *Curvularia* leaf blight disease was observed in three farms of *Curcuma aromatica* Salisb. in Hainan Province during 2010 (Chen et al. 2013). Gao et al. (2012) reported a new rice black sheath spot disease caused by *C. fallax* Boedijn in Hunan Province. Our research group is also conducting a disease survey on the occurrence of *Curvularia* diseases in Southwest China since 2017. Two new pathogens (*C. asianensis* Manamgoda, L. Cai & K.D. Hyde and *C. microspora* Y. Liang, K.D. Hyde, J. Bhat & Yong Wang bis), which affected *Epipremnum pinnatum* (L.) Engl. and *Hippeastrum rutilum* Herb. (Liang et al. 2018; Wang et al. 2018), respectively, were found.

Meanwhile, a severe leaf blast disease on lemongrass was found in Guangxi Province, China, that first appeared on the tips of leaves. As the infection progressed, more than 30% of leaves showed different degrees of abnormalities, while in the later stages more than 50% of the upper leaves appeared diseased and disease incidence reached 80% or above in the lower leaf blades. We provide a detailed morphological description and phylogenetic analyses of the pathogen confirming it as a new *Curvularia* species. Koch’s postulates (see later text) have been carried out to confirm its pathogenicity. Our study provides a further understanding of *Curvularia* disease on lemongrass in China.

## Materials and methods

### Isolation

Leaves of *Cymbopogon citratus* showing leaf blast symptoms were collected from Guangxi Medicinal Botanical Garden in Nanning, China, during 2017. Diseased leaf pieces were surface disinfected with 70% ethanol for 30 s, 1% NaClO for 1 min and repeatedly rinsed in sterile distilled water for 30 s. For isolation of *Curvularia*, conidia were removed from the diseased tissue surface using a sterilised needle and placed in a drop of sterilised water followed by microscopic examination. The spore suspension was drawn with a Pasteur pipette and transferred to a Petri dish with 2% water agar (WA) or 2% malt extract agar (MEA) and 100 mg/l streptomycin to inhibit the growth of bacteria. The plates were incubated for 24 h in an incubator (25°C) and examined for single spore germination under a dissecting microscope. Germinating conidia were transferred separately to new 2% MEA plates (Chomnunti et al. 2014).

### Morphological studies

Single germinated spores were transferred to PDA or MEA and incubated at 28°C in a light incubator with 12 h light/12 h darkness. Ten days later, the colony and morphological characters were recorded according to Manamgoda et al. (2011, 2012). Colony diameters on PDA and MEA were measured at 1, 3, 5 and 7 days post-inoculation and average growth rates were calculated. Conidia and conidiophores were examined using a compound microscope fitted with a digital camera (Olympus BX53). The holotype specimen is deposited in the Herbarium of the Department of Plant Pathology, Agricultural College, Guizhou University (HGUP). An ex-type culture is deposited in the Culture Collection of the Department of Plant Pathology, Agriculture College, Guizhou University, China (GUCC) and Mae Fah Luang University Culture Collection (MFLUCC) in Thailand (Table 1).

**Table 1.** Sequences used for phylogenetic analysis.

**Species name**	**Strain number**	**GenBank Accession numbers**		
		**ITS**	**GAPDH**	**tef1**
*Curvularia aeria*	CBS 294.61^T^	HE861850	HF565450	–
*C. affinis*	CBS 154.34^T^	KJ909780	KM230401	KM196566
*C. ahvazensis*	CBS 144673^T^	KX139029	MG428693	MG428686
*C. akaii*	CBS 317.86	KJ909782	KM230402	KM196569
*C. akaiiensis*	BRIP 16080^T^	KJ415539	KJ415407	KJ415453
*C. alcornii*	MFLUCC 10-0703^T^	JX256420	JX276433	JX266589
*C. americana*	UTHSC 08-3414^T^	HE861833	HF565488	–
*C. asiatica*	MFLUCC 10-0711^T^	JX256424	JX276436	JX266593
*C. australiensis*	BRIP 12044^T^	KJ415540	KJ415406	KJ415452
*C. australis*	BRIP 12521^T^	KJ415541	KJ415405	KJ415451
*C. bannonii*	BRIP 16732^T^	KJ415542	KJ415404	KJ415450
*C. beasleyi*	BRIP 10972^T^	MH414892	MH433638	MH433654
*C. beerburrumensis*	BRIP 12942^T^	MH414894	MH433634	MH433657
*C. boeremae*	IMI 164633^T^	MH414911	MH433641	–
*C. borreriae*	CBS 859.73	HE861848	HF565455	–
	MFLUCC 11-0422	KP400638	KP419987	KM196571
*C. bothriochloae*	BRIP 12522^T^	KJ415543	KJ415403	KJ415449
*C. brachyspora*	CBS 186.50	KJ922372	KM061784	KM230405
*C. buchloes*	CBS 246.49^T^	KJ909765	KM061789	KM196588
*C. carica-papayae*	CBS 135941^T^	HG778984	HG779146	–
*C. chiangmaiensis*	CPC 28829^T^	MF490814	MF490836	MF490857
*C. chlamydospora*	UTHSC 07-2764^T^	HG779021	HG779151	–
*C. clavata*	BRIP 61680b	KU552205	KU552167	KU552159
*C. coatesiae*	BRIP 24261^T^	MH414897	MH433636	MH433659
*C. coicis*	CBS 192.29^T^	JN192373	JN600962	JN601006
*C. colbranii*	BRIP 13066^T^	MH414898	MH433642	MH433660
*C. crustacea*	BRIP 13524^T^	KJ415544	KJ415402	KJ415448
*C. cymbopogonis*	CBS 419.78	HG778985	HG779129	–
*C. dactyloctenicola*	CPC 28810^T^	MF490815	MF490837	MF490858
*C. dactyloctenii*	BRIP 12846^T^	KJ415545	KJ415401	KJ415447
*C. deightonii*	CBS 537.70	LT631356	LT715839	–
*C. ellisii*	CBS 193.62^T^	JN192375	JN600963	JN601007
*C. eragrosticola*	BRIP 12538^T^	MH414899	MH433643	MH433661
*C. eragrostidis*	CBS 189.48	HG778986	HG779154	–
*C. geniculata*	CBS 187.50^T^	KJ909781	KM083609	KM230410
*C. gladioli*	CBS 210.79	HG778987	HG779123	
*C. graminicola*	BRIP 23186^T^	JN192376	JN600964	JN601008
*C. gudauskasii*	DAOM 165085	AF071338	–	–
*C. harveyi*	BRIP 57412^T^	KJ415546	KJ415400	KJ415446
*C. hawaiiensis*	BRIP 11987^T^	KJ415547	KJ415399	KJ415445
*C. heteropogonicola*	BRIP 14579^T^	KJ415548	KJ415398	KJ415444
*C. heteropogonis*	CBS 284.91^T^	JN192379	JN600969	JN601013
*C. hominis*	CBS 136985^T^	HG779011	HG779106	–
*C. homomorpha*	CBS 156.60^T^	JN192380	JN600970	JN601014
*C. inaequalis*	CBS 102.42^T^	KJ922375	KM061787	KM196574
*C. intermedia*	CBS 334.64	HG778991	HG779155	–
*C. ischaemi*	CBS 630.82^T^	JX256428	JX276440	–
*C. kenpeggii*	BRIP 14530^T^	MH414900	MH433644	MH433662
*C. kusanoi*	CBS 137.29^T^	JN192381	–	JN601016
*C. lamingtonensis*	BRIP 12259^T^	MH414901	MH433645	MH433663
*C. lunata*	CBS 730.96^T^	JX256429	JX276441	JX266596
*C. malina*	CBS 131274^T^	JF812154	KP153179	KR493095
*C. mebaldsii*	BRIP 12900^T^	MH414902	MH433647	MH433664
*C. micropus*	CBS 127235^T^	HE792934	LT715859	–
*C. microspora*	GUCC 6272^T^	MF139088	MF139106	MF139115
*C. miyakei*	CBS 197.29^T^	KJ909770	KM083611	KM196568
*C. mosaddeghii*	IRAN 3131C^T^	MG846737	MH392155	MH392152
*C. muehlenbeckiae*	CBS 144.63^T^	HG779002	HG779108	–
*C. neergaardii*	BRIP 12919^T^	KJ415550	KJ415397	KJ415443
*C. nanningensis* sp. nov.	GUCC 11000	MH885316	MH980000	MH980006
	GUCC 11001	MH885317	MH980001	MH980007
	GUCC 11002	MH885318	MH980002	MH980008
	GUCC 11003	MH885319	MH980003	MH980009
	GUCC 11005^T^	MH885321	MH980005	MH980011
*C. neoindica*	BRIP 17439	AF081449	AF081406	–
*C. nicotiae*	CBS 655.74^T^ = BRIP 11983	KJ415551	KJ415396	KJ415442
*C. nodosa*	CPC 28800^T^	MF490816	MF490838	MF490859
	CPC 28801	MF490817	MF490839	MF490860
	CPC 28812	MF490818	MF490840	MF490861
*C. nodulosa*	CBS 160.58	JN601033	JN600975	JN601019
*C. oryzae*	CBS 169.53^T^	KP400650	KP645344	KM196590
*C. ovariicola*	CBS 470.90^T^	JN192384	JN600976	JN601020
*C. pallescens*	CBS 156.35^T^	KJ922380	KM083606	KM196570
*C. palmicola*	MFLUCC 14-0404	MF621582	–	–
*C. papendorfii*	CBS 308.67^T^	KJ909774	KM083617	KM196594
*C. perotidis*	CBS 350.90^T^	JN192385	KJ415394	JN601021
*C. petersonii*	BRIP 14642^T^	MH414905	MH433650	MH433668
*C. pisi*	CBS 190.48^T^	KY905678	KY905690	KY905697
*C. platzii*	BRIP 27703b^T^	MH414906	MH433651	MH433669
*C. portulacae*	CBS 239.48^T^ = BRIP 14541	KJ415553	KJ415393	KJ415440
*C. prasadii*	CBS 143.64^T^	KJ922373	KM061785	KM230408
*C. protuberata*	CBS 376.65^T^	KJ922376	KM083605	KM196576
*C. pseudobrachyspora*	CPC 28808^T^	MF490819	MF490841	MF490862
*C. pseudolunata*	UTHSC 09-2092^T^	HE861842	HF565459	–
*C. pseudorobusta*	UTHSC 08-3458	HE861838	HF565476	–
*C. ravenelii*	BRIP 13165^T^	JN192386	JN600978	JN601024
*C. reesii*	BRIP 4358^T^	MH414907	MH433637	MH433670
*C. richardiae*	BRIP 4371^T^	KJ415555	KJ415391	KJ415438
*C. robusta*	CBS 624.68^T^	KJ909783	KM083613	KM196577
*C. rouhanii*	CBS 144674^T^	KX139030	MG428694	MG428687
*C. ryleyi*	BRIP 12554^T^	KJ415556	KJ415390	KJ415437
*C. senegalensis*	CBS 149.71	HG779001	HG779128	–
*C. sesuvii*	Bp-Zj 01^T^	EF175940	–	–
*C. shahidchamranensis*	IRAN 3133C^T^	MH550084	MH550083	–
*C. soli*	CBS 222.96^T^	KY905679	KY905691	KY905698
*C. sorghina*	BRIP 15900^T^	KJ415558	KJ415388	KJ415435
*C. spicifera*	CBS 274.52	JN192387	JN600979	JN601023
*C. sporobolicola*	BRIP 23040b^T^	MH414908	MH433652	MH433671
*C. subpapendorfii*	CBS 656.74^T^	KJ909777	KM061791	KM196585
*C. trifolii*	CBS 173.55	HG779023	HG779124	–
*C. tripogonis*	BRIP 12375^T^	JN192388	JN600980	JN601025
*C. tropicalis*	BRIP 14834^T^	KJ415559	KJ415387	KJ415434
*C. tsudae*	ATCC 44764^T^	KC424596	KC747745	KC503940
*C. tuberculata*	CBS 146.63^T^	JX256433	JX276445	JX266599
*C. uncinata*	CBS 221.52^T^	HG779024	HG779134	–
*C. variabilis*	CPC 28813	MF490820	MF490842	MF490863
	CPC 28814	MF490821	MF490843	MF490864
	CPC 28815^T^	MF490822	MF490844	MF490865
	CPC 28816	MF490823	MF490845	MF490866
*C. verruciformis*	CBS 537.75	HG779026	HG779133	–
*C. verruculosa*	CBS 150.63	KP400652	KP645346	KP735695
	CPC 28792	MF490825	MF490847	MF490868
	CPC 28809	MF490824	MF490846	MF490867
*C. warraberensis*	BRIP 14817^T^	MH414909	MH433653	MH433672
*Bipolaris drechsleri*	MUS0028	KF500532	KF500535	KM093761
*B. maydis*	CBS 136.29^T^	AF071325	KM034846	KM093794

Ex-type isolates were labeled with “^T^”.

### DNA Extraction and Sequencing

Fungal cultures were grown on PDA at 28°C until the entire Petri dish (90 mm) was colonised. Fresh fungal mycelia were scraped off the surface of the PDA using a sterilised scalpel. A BIOMIGA Fungus Genomic DNA Extraction Kit (GD2416, BIOMIGA, Inc., San Diego, California, USA) was used to extract the genomic DNA. DNA amplification was performed in a 25 μl reaction volume which contained 2.5 μl 10 × PCR buffer, 1 μl of each primer (10 μM), 1 μl template DNA, 0.25 μl Taq DNA polymerase (Promega, Madison, WI, USA) and 18.5 μl ddH_2_O. Primers used and thermal cycling programme for PCR amplification of the ITS (ITS4/ITS5), GAPDH (gpd1/gpd2) and *tef*1 (EF-526F/1567R) genes were followed as described previously (White et al. 1990; Berbee et al. 1999; Schoch et al. 2009; Liang et al. 2018).

### Phylogenetic analyses

DNA sequences originated from five strains (GUCC 11000, GUCC 11001, GUCC 11002, GUCC 11003 and GUCC 11005) and reference sequences of ex-type or representative sequences of *Curvularia* species were downloaded from GenBank database (Table 1) with strains of *Bipolaris maydis* (Y. Nisik. & C. Miyake) Shoemaker (CBS 136.29) and *B. drechsleri* Manamgoda & Minnis (MUS0028) as outgroup taxa. Alignments for each locus were performed in MAFFT v7.307 online version (Katoh and Standley 2016) and manually verified in MEGA 6.06 (Tamura et al. 2013). Phylogenetic analyses were performed by Maximum Parsimony (MP), Maximum Likelihood (ML) and Bayesian methods. Sequences were optimised manually to allow maximum alignment and maximum sequence similarity as detailed in Manamgoda et al. (2012). MP analyses were performed in PAUP v. 4.0b10 (Swofford 2003) using the heuristic search option with 1,000 random taxa additions and tree bisection and reconnection (TBR) as the branch-swapping algorithm. Five thousand maxtrees were set to build up the phylogenetic tree. The characters in the alignment matrix were ordered according to ITS+GAPDH+*tef*1 with equal weight, and gaps were treated as missing data. The MP phylogenetic analysis of *Curvularia* ITS sequences included pathogens from China, India and Pakistan and the wrong sequence (KN879930), actually belonging to *Alternaria alternata* (taxon:5599), was selected as the outgroup. The Tree Length (TL), Consistency Indices (CI), Retention Indices (RI), Rescaled Consistency Indices (RC) and Homoplasy Index (HI) were calculated for each tree generated. The resulting PHYLIP file was used to generate the ML tree on the CIPRES Science Gateway (https://www.phylo.org/portal2/login.action) using the RAxML-HPC2 black box with 1000 bootstrap replicates and GTRGAMMA as the nucleotide substitution model. For Bayesian inference analysis, the best model of evolution (GTR+I+G) was determined using MrModeltest v2 (Nylander 2004). Bayesian inference analysis was done using MrBayes v 3.2.6 (Ronquist et al. 2012). Bayesian analyses were launched with random starting trees for 2 000 000 generations and Markov chains were sampled every 1000 generations. The first 25% resulting trees were discarded as burn-in. Alignment matrices are available in TreeBASE under the study ID 25080.

### Koch’s Postulate test

To confirm the pathogenicity of the fungus, five healthy plants of *Cymbopogon citratus* were inoculated with 5 mm diameter mycelial plugs of the five isolates (GUCC 11000, GUCC 11001, GUCC 11002, GUCC 11003 and GUCC 11005) cut from the margins of 10-day-old actively growing cultures; the control was treated with sterile agar plugs. The plants were kept for two days in an illuminating incubator at 28° ± 3°C. Additionally, two plants were sprayed with distilled water and kept as control under the same conditions. Both inoculated (host and detached leaves) and control plants were kept for two days in an illuminating incubator at 28 ± 3°C. After four days of incubation, the inoculated plants and leaves were observed for the development of symptoms (Zhang et al. 2018). Infected leaves were collected and the fungus was re-isolated using PDA medium and the ITS sequence was compared with original strains.

## Results

### Phylogenetic analyses

First, we compared the DNA sequence identity of ITS, GAPDH and *tef*1 gene regions (Table 2). Among our five strains, there was only one base difference. In the ITS gene region, for *C. akaiiensis*, the base sequence was identical to our strains; only 1 difference for *C. bothriochloae*; base differences were 8 for *C. akaii*, 9 for *C. deightonii* and 5 for *C. sichuanensis*. Only *C. heteropogonis* had noticeable (25) base differences with our strains. In the GAPDH and *tef*1 gene regions, the mutation rate of DNA bases was apparently faster than the ITS region. There were between 9 to 19 base differences in GAPDH and 3 to 8 in *tef*1. This means that in *Curvularia*, GAPDH has a faster evolutionary rate than ITS and *tef*1 and therefore some mycologists have suggested the use of ITS+GAPDH for phylogenetic analysis and GAPDH as a secondary barcode marker for accurate identification.

**Table 2.** DNA sequence differences between *Curvularia nanningensis* and related species in three gene regions.

**Species**	**Strain number**	**ITS (1–547 bp)**	**GAPDH (550–1031bp)**	**tef1 (1034–1899 bp)**
*C. nanningensis*	GUCC11000	0	1	0
	GUCC11001	0	0	0
	GUCC11002	0	1	0
	GUCC11003	0	1	0
	GUCC11005^T^	0	0	0
*C. akaii*	CBS 317.86	8	9	4
*C. akaiiensis*	BRIP 16080 ^T^	0	10	5
*C. bothriochloae*	BRIP 12522 ^T^	1	19	8
*C. deightonii*	CBS 537.70	9	13	–
*C. heteropogonis*	CBS 284.91 ^T^	25	12	3
*C. sichuanensis*	HSAUP II.2650-1 ^T^	5	–	–

^T^ = ex-type

**Figure 1.**
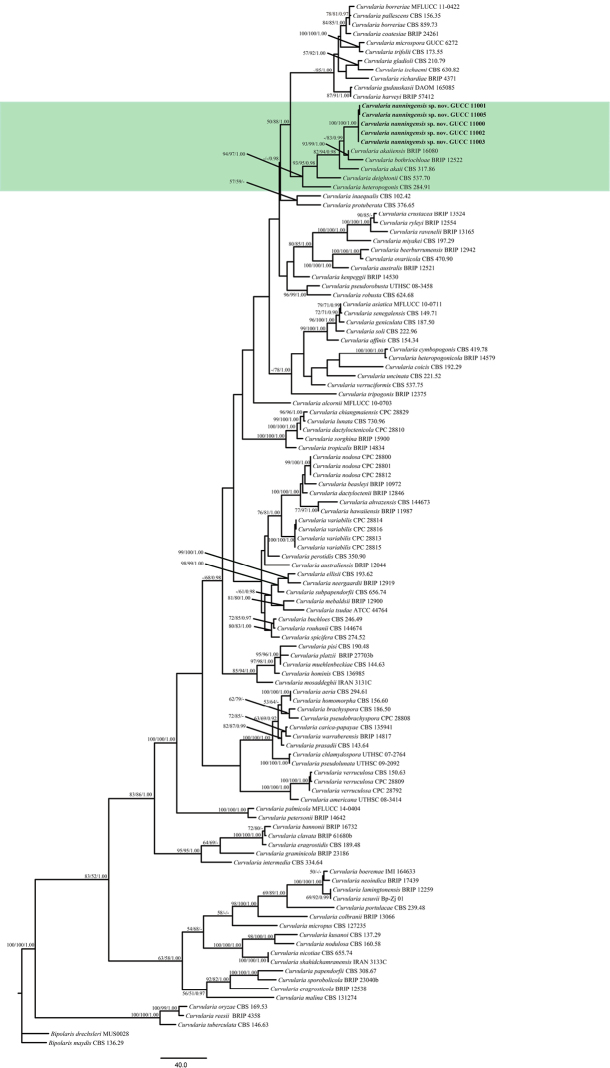
Maximum Parsimony (MP) topology of *Curvularia* generated from a combination of ITS, GAPDH and *tef*1 sequences. *Bipolaris maydis* (CBS 136.29) and *B. drechsleri* (MUS0028) were used as outgroup taxa. MP and ML above 50% and BPP values above 0.90 were placed close to topological nodes and separated by “/”. The bootstrap values below 50% and BPP values below 0.90 were labelled with “-”. Our main research clade was labelled with green colour.

The alignment of *Curvularia* combining three gene fragments (ITS, GAPDH and *tef*1) comprised 116 strains belonging to 104 taxa. In order to accurately identify our strains, phylogenetic analysis included all ex-type and published strains of all *Curvularia* spp. described recently (Hyde et al. 2017; Marin-Felix et al. 2017; Dehdari et al. 2018; Heidari et al. 2018; Hernández-Restrepo et al. 2018; Mehrabi-Koushki et al. 2018; Tan et al. 2018; Jayawardena et al. 2019) which are listed in Table 1. The final alignment comprised 2032 characters (each gene fragment was separated with 2 “N”) including gaps (ITS: 1−600, GAPDH: 603−1162 and tef1: 1165−2032). Among these characters, 2032 are constant, 125 variable characters are parsimony-uninformative and 503 are parsimony-informative. The parameters of the phylogenetic trees are TL = 2590, CI = 0.38, RI = 0.72 and HI = 0.62. In the *Curvularia* phylogenetic tree (Figure 1), all isolates grouped together with 100% (MP and ML) bootstrap support. Our strains (GUCC 11000, 11001, 11002, 11003 and 11005) formed a strongly supported group (MP: 100%; ML: 100%; BPP: 1.00) with a close relationship to *C. akaii*, *C. akaiiensis*, *C. bothriochloae*, *C. deightonii* and *C. heteropogonis* with high bootstrap support (MP: 94%; ML: 97%; BPP: 1.00). In this group, the five examined strains were closer to *C. akaii*, *C. akaiiensis* and *C. bothriochloae* and also showed high bootstrap support (MP: 82% and ML: 94%; BPP: 0.98).

**Figure 2.**
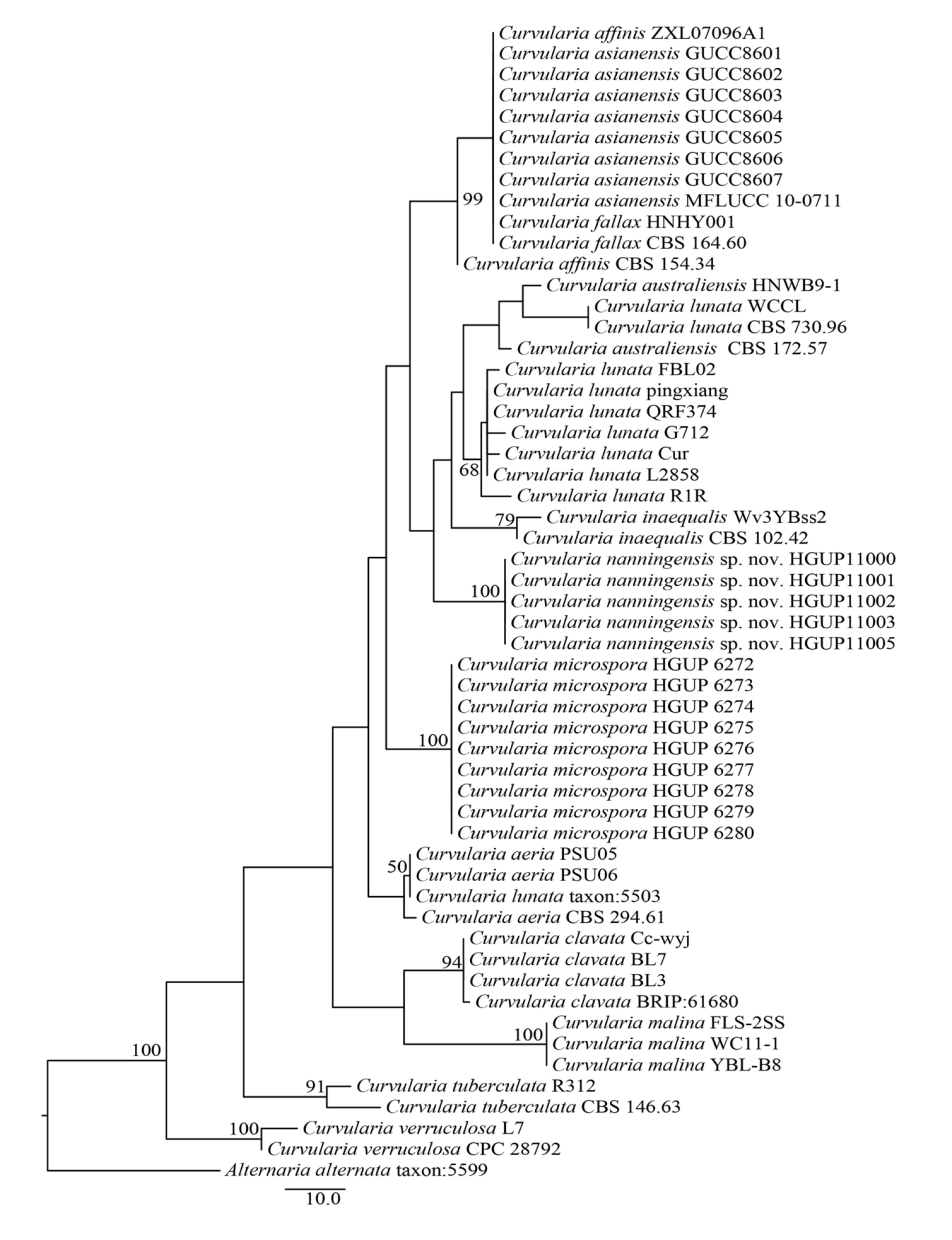
Maximum Parsimony (MP) analysis of *Curvularia* pathogens in China, India and Pakistan based on ITS sequences. *Alternaria alternata* (taxon:5599) was used as outgroup taxon. Bootstrap values (≥ 50%) of the MP method are shown near the nodes.

The phylogenetic analysis of the ITS gene region evaluated all new *Curvularia* pathogens recently described from China, India and Pakistan. The aligned matrix consisted of fifty-four ITS sequences and included ex-type sequences of 13 *Curvularia* species (Supplementary Table 1). The phylogenetic tree (Figure 2) indicated that ITS BLAST searches only provided limited value for pathogenic identification. In *Curvularia lunata*, only one sequence WCCL (MG063428) showed a very close relationship with the ex-type strain sequence of *C. lunata* CBS 730.96 (MG722981). The other eight sequences were grouped into two branches, e.g. taxon:5503 (LN879926) which might belong to *C. aeria*, while the other seven formed an independent lineage. ITS sequences did not separate *Curvularia affinis*, *C. asianensis* and *C. fallax* and some of their sequences even clustered with *C. australiensis* HNWB9-1 (KT719300). After multi-gene analysis, the phylogenetic distance was shown to be unreliable and may suggest whether they belong perhaps to different species.

### Taxonomy

#### Curvularia nanningensis

Taxon classificationFungiPoalesPoaceae

Qian Zhang, K.D. Hyde & Yong Wang bis sp. nov.

A4D38981-18A5-5CDC-8EFD-78FDF5F429CF

829056

Figure 3A–I

##### Diagnosis.

Characterised by the size of conidia.

##### Type.

China, Guangxi Province, Nanning City, Guangxi Medicinal Botanical Garden, 22°51’N, 108°19’E, on blighted leaves of *Cymbopogon citratus*, 30 September 2017, Q. Zhang, ZQ0091 (HGUP 11005, holotype, MFLU19-1227, isotype), GUCC 11005 and MFLUCC 19-0092, ex-type.

##### Description.

Pathogenic on *Cymbopogon citratus*. Fungus initially producing white to grey lesions with dark borders on all parts of the shoot, later enlarging and coalescing over entire leaf.

*Colonies* on PDA irregularly circular, with mycelial growth rate = 1.0 cm/day, vegetative hyphae septate, branched, subhyaline to brown, smooth to verruculose, 2–3 µm, anastomosing. *Aerial mycelium* dense, felted, initially pale grey, becoming darkened and greyish-green at maturity, producing black extracellular pigments. On MEA, the colony morphology similar to PDA, with growth rate = 1.35 cm/day. **Sexual morph**: Undetermined. **Asexual morph**: Hyphomycetous. *Conidiophores* macronematous, arising singly, simple or branched, flexuous, 8–10 septate, geniculate, pale brown to dark brown, paler towards apex, 120–200 × 2–3 µm (av. = 170 × 2.5 µm, n = 30). *Conidiogenous cells* polytretic, sympodial, terminal, sometimes intercalary, cicatrised, with thickened and darkened conidiogenous loci up to 1.0–1.2 µm diam., smooth. *Mature conidia* 3 to rarely 4 septa, acropleurogenous, obovoid, usually straight to curved at the slightly wider, smooth-walled, larger third cell from the base, 24.5–36.0 × 14.0–20.5 µm (av. = 29.5 × 17.5 µm, n = 50), sub-hyaline to pale brown end cells, pale brown to dark brown at intermediate cells, with conspicuous or sometimes slightly protuberant hilum. Germination of conidia bipolar.

##### Distribution.

China, Guangxi Province, Nanning City.

##### Other material examined.

China, Guangxi Province, Nanning city, Guangxi Medicinal Botanical Garden, on blight leaves of *C. citratus*, 30 September 2017, Q. Zhang, ZQ0087 (HGUP 11000); ZQ0088 (HGUP 11001); ZQ0089 (HGUP 11002); ZQ0090, (HGUP 11003).

##### Etymology.

With reference to the location, Nanning City where the fungus was isolated.

**Figure 3.**
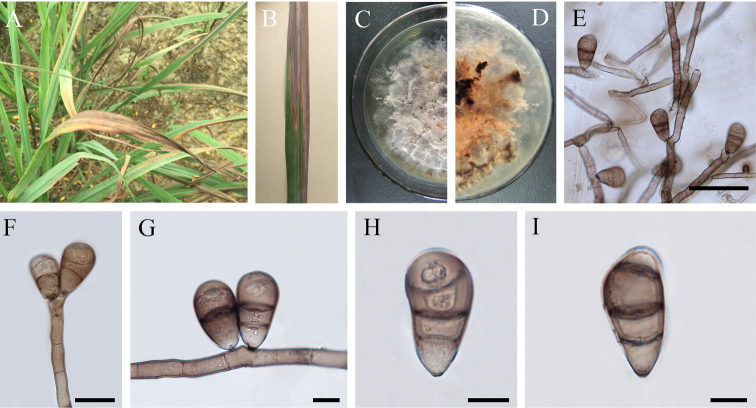
*Curvularia nanningensis* (GUCC11005, holotype) **A**, **B** diseased symptom **C** colony on PDA from above **D** colony on PDA from below **E**−**G** conidia and conidiophores **H**−**I** conidia. Scale bars: 50 μm (**E**), 20 μm (**F**), 10 μm (**G**−**I**).

### Pathogenicity test

**Figure 4.**
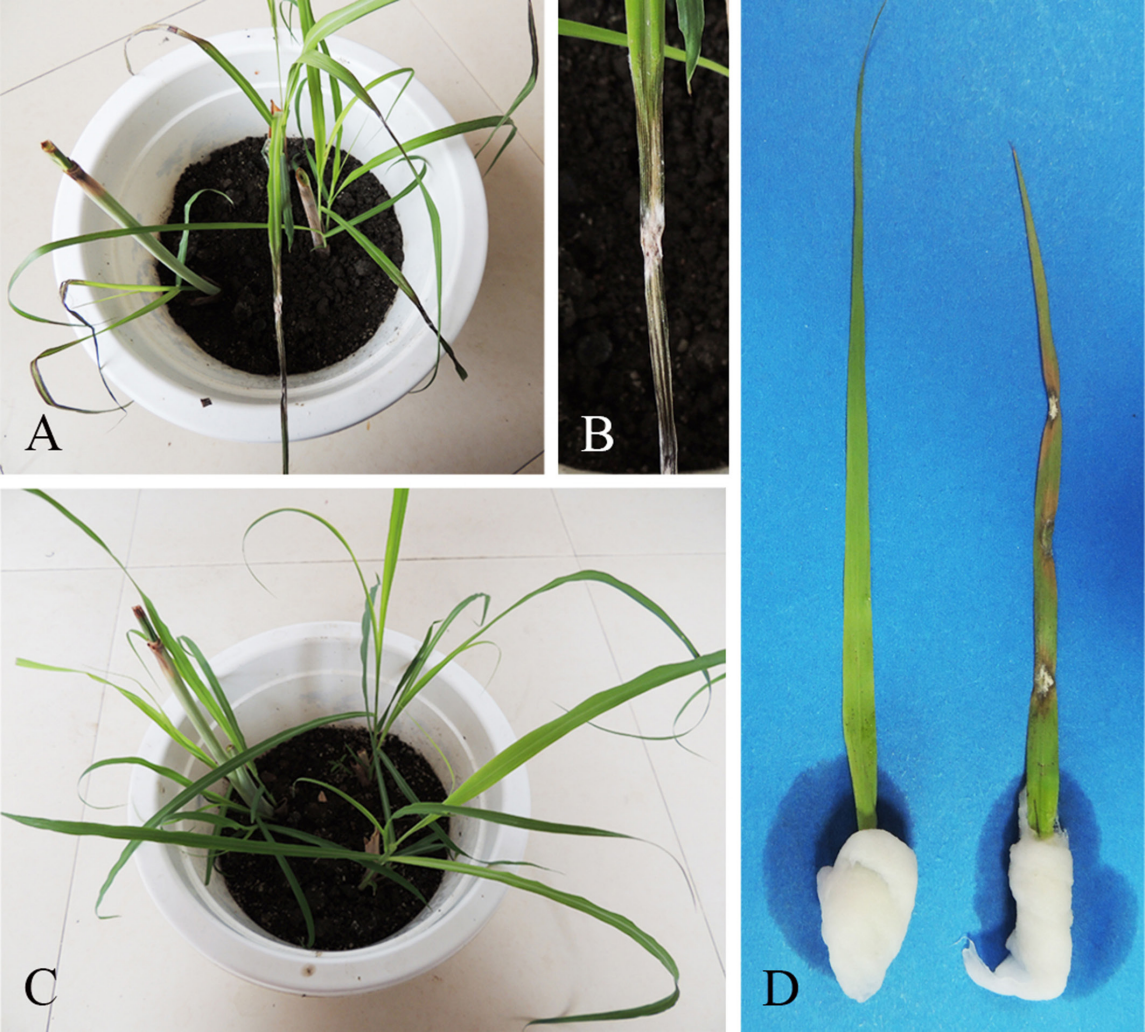
Pathogen inoculation and symptom (4 days). **A***Cymbopogon citratus* inoculated and disease symptom **B** inoculation point and disease symptom **C** control **D** detached experiment. Left. Control. Right. Inoculation point and disease symptoms.

Four days after inoculation, blast symptoms appeared on all inoculated plants, which were similar to symptoms of plants in the field (Figures 3A, B, 4A, B). Non-treated control plants remained healthy without any symptoms (Figure 4C). *Curvularia nanningensis* was re-isolated from the lesions of inoculated plants and the identity of the fungus was confirmed by sequencing the ITS region. Meanwhile, a detached leaf-experiment was also conducted in an illuminated incubator at 28 ± 3°C, where similar symptoms appeared on healthy inoculated leaves of *Cymbopogon citratus* after four days (Figure 4 D right), while the control leaf (Figure 4 D left) did not show symptoms.

## Discussion

Phylogenetic analysis based on combined DNA sequences of ITS, GAPDH and *tef*1 showed that our strains were related to three *Curvularia* species named *C. akaii* (Tsuda & Ueyama) Sivan., *C. akaiiensis* Sivan. and *C. bothriochloae* Sivan., Alcorn & R.G. Shivas. The main morphological characters that discriminate our strains from related species are the size-range of conidia and length of conidiophores. *Curvularia bothriochloae* produced conidia measuring 30–47 × 15–25 µm (Sivanesan et al. 2003) while *C. akaiiensis* produced the smallest conidia (22.5–27.5 × 7.5–15.5 µm). Conidial length of *C. nanningensis* was very close to *C. akaii* (24–34 µm) (Tsuda and Ueyama 1985) but the conidia of our species were broader than those of *C. akaii* (8.7–13.8 µm). Conidiophores of *C. nanningensis* were shorter than those of *C. bothriochloae* (360–425 µm) (Alcorn 1990). In the case of *C. sichuanensis* Meng Zhang & T.Y. Zhang, only one ITS sequence AB453881 was available in GenBank for analysis. While examining our sequences, only 4–5 bp differences were revealed in 499 bp characters between *C. nanningensis* and *C. sichuanensis*, thus indicating a close relationship between the two strains based on ITS sequence data and likely between the two species. However, according to Zhang et al. (2007), the conidial width of *C. sichuanensis* (10–15 µm) is smaller than *C. nanningensis* (14–20.5 µm) on PDA. For *C. sichuanensis*, the conidial wall of the median cell is deepened and thickened while *C. nanningensis* obviously does not have these characters. Meanwhile, the hilum of conidia in *C. sichuanensis* is obviously protuberant while *C. nanningensis* lacked this character.

The pathogenicity test based on natural inoculation and detached leaves (Figure 3) confirmed that *Curvularia nanningensis* is a pathogen of *Cymbopogon citratus* blast disease. We previously named our strains as *C. cymbopogonis* following a previous report of the species by Groves and Skolko (1945) as a seed-borne pathogen of *Cymbopogon nardus*. *Curvularia cymbopogonis* is a common pathogen which also causes diseases of sugar-cane, rice, seedlings of itchgrass, *Agrostis palustris* Huds. and *Dactylis glomerata* L. (Santamaria et al. 1971; Walker and White 1979; Olufolaji 1996; Yi et al. 2002). A single strain named *C. cymbopogonis* (CBS 419.78) included in our analyses grouped distant from *C. nanningensis* but its reliability seems questionable and apparently belongs to a different species (Fig. 1). We further checked the original description of this species (Groves and Skolko 1945) and found that differences in conidial shape mainly resulted from conidial width (*C. cymbopogonis*: 11–13 µm vs *C. nanningensis*: 14–20.5 µm). Additionally, Groves and Skolko (1945), Hall and Sivanesan (1972) and Yi et al. (2002) reported that *C. cymbopogonis* produced 4 to 5-septate conidia, whereas conidia of *C. nanningensis* only had 3-septa. *Curvularia* spp. are important pathogens of lemongrass. Morphological studies together with phylogenetic analyses provided evidence that *C. nanningensis* is a new pathogen distinct from all hitherto reported diseases on lemongrass. Our findings expanded the documented diversity of *Cymbopogon* pathogens within the genus *Curvularia* and further clarified the taxonomy of this novel pathogen, *Curvularia nanningensis*.

Moreover, 29 first reports of *Curvularia* diseases on different plants in China, India and Pakistan were found in the literature from 2010 to the present. It is evident that in this vast geographical area, *Curvularia* spp. have maintained a close association with plant diversity and thereby possess a rich fungal diversity that is affected by crops distribution. Among them, six reports only provided morphological data and more than half (16) only referred to ITS sequence data and morphological description (Suppl. Table 1). For unknown reasons, Iftikhar et al. (2016) misidentified the *Curvularia* pathogen with an *Alternaria* sequence (LN879930.1). Our phylogenetic tree, based on 54 reported ITS sequence data of *Curvularia* diseases in these countries (Figure 2), also indicated that this approach is not effective for identifying these pathogens, especially in the case of *C. lunata* as a prevalent species. However, identification of *Curvularia* isolates by multi-gene phylogenetic analyses has withstood scrutiny (Liang et al. 2018; Wang et al. 2018; Zhang et al. 2018). Additionally, nearly all reports, even for severe diseases, are based on a single isolate, which preclude an objective evaluation. We, therefore, propose the following standardised steps as required for the reliable identification of *Curvularia* diseases: 1) collect several isolates from diseased samples, 2) obtain sequences of the ITS, GAPDH and *tef*1 or at least ITS+GAPDH for phylogenetic analysis, 3) perform BLAST searches with sequences originated from ex-type or representative strains in GenBank, and 4) combine morphological comparison and phylogenetic analysis for accurate identification.

## Supplementary Material

XML Treatment for Curvularia nanningensis

## Supplementary materials

Supplementary material 1 Table S1 Data type: (measurement/occurrence/multimedia/etc.) https://binary.pensoft.net/file/380726This dataset is made available under the Open Database License (http://opendatacommons.org/licenses/odbl/1.0/). The Open Database License (ODbL) is a license agreement intended to allow users to freely share, modify, and use this Dataset while maintaining this same freedom for others, provided that the original source and author(s) are credited.Qian Zhang, Zai-Fu Yang, Wei Cheng, Nalin N. Wijayawardene, Kevin D. Hyde, Zhuo Chen, Yong Wang
