# Supplementary material for: Diseases of Cymbopogon citratus (Poaceae) in China: Curvularia nanningensis sp. nov
Source: MycoKeys. 2020 Feb 13;63:49–67. doi: 10.3897/mycokeys.63.49264 (PMC7033261; doi:10.3897/mycokeys.63.49264)
Supplement: Supplementary material 1 [file mycokeys-63-049-s001.doc]

Sul Table 1 Disease occurrence caused by *Curvularia* spp. in China, India and Pakistan.

| Species name | Strain number | ITS | References |
| --- | --- | --- | --- |
| *Curvularia aeria* | PSU05 | LC314151 | Pornsuriya C, Ito SI, Sunpapao A (2018) First report of leaf spot on lettuce caused by *Curvularia aeria*. Journal of General Plant Pathology 84(4): 296–299. |
| *Curvularia aeria* | PSU06 | LC314152 |
| *Curvularia aeria* | CBS 294.61 | MH858056 | Vu D, Groenewald M, De VM , et al. (2019) Large-scale generation and analysis of filamentous fungal DNA barcodes boosts coverage for kingdom fungi and reveals thresholds for fungal species and higher taxon delimitation. Studies in Mycology 92: 135–154. |
| *Curvularia affinis* | ZXL07096A1 | GU073105 | unpublished |
| *Curvularia affinis* | CBS 154.34 | MH855475 | Vu D, Groenewald M, De VM , et al. (2019) Large-scale generation and analysis of filamentous fungal DNA barcodes boosts coverage for kingdom fungi and reveals thresholds for fungal species and higher taxon delimitation. Studies in Mycology 92: 135–154. |
| *Curvularia affinis* |  | no sequence | Sharma P, Singh N, Verma OP (2012) First report of *Curvularia* leaf spot, caused by *Curvularia affinis* on *Dalbergia sissoo*. Forest Pathology 42(3): 265–266. |
| *Curvularia asianensis* | GUCC8601 | MG734378 | Wang Y, Pan XJ, Zhang Q, Zhao DG (2018). First report of *Curvularia asianensis*, a leaf blotch disease associated with *Epipremnum pinnatum* in Guangxi Autonomous Region of China. Plant Disease 102(9): 1854. |
| *Curvularia asianensis* | GUCC8602 | MG734379 |
| *Curvularia asianensis* | GUCC8603 | MG734380 |
| *Curvularia asianensis* | GUCC8604 | MG734381 |
| *Curvularia asianensis* | GUCC8605 | MG734382 |
| *Curvularia asianensis* | GUCC8606 | MG734383 |
| *Curvularia asianensis* | GUCC8607 | MG734384 |
| *Curvularia asianensis* | MFLUCC 10-0711 | JX256424 | Manamgoda DS, Cai L, Mckenzie E H C, et al. (2012) A phylogenetic and taxonomic re-evaluation of the *Bipolaris*-*Cochliobolus*-*Curvularia* complex. Fungal Diversity 56(1):131–144. |
| *Curvularia australiensis* | HNWB9-1 | KT719300 | Chang JY, Zhang HJ, Shi J, Guo N, Hu QY, Ma HX, Yang S (2016) First Report of *Curvularia australiensis* causing leaf spot on maize (*Zea mays*) in China. Plant Disease 100(8): 1780. |
| *Curvularia australiensis* | CBS 172.57 | MH857686 | Vu D, Groenewald M, De VM , et al. (2019) Large-scale generation and analysis of filamentous fungal DNA barcodes boosts coverage for kingdom fungi and reveals thresholds for fungal species and higher taxon delimitation. Studies in Mycology 92: 135–154. |
| *Curvularia clavata* | NFCCI 2673 | no sequence | Narmadhavathy S, Vanitha S, Karthikeyan G, Raguchander T, Ramjegathesh R (2013) First report of leaf blight of physic nut caused by *Curvularia clavata* in India. Journal of Plant Pathology 95(3): 659. |
| *Curvularia clavata* | Cc-wyj | JQ730852 | Chen XY, Feng JD, Su Z, et al. (2013) First report of Curvularia leaf blight on *Curcuma wenyujin* caused by *Curvularia clavata* in China. Plant Disease 97(1): 138. |
| *Curvularia clavata* | BL7 | KP692788 | unpublished |
| *Curvularia clavata* | BL3 | KP700959 | unpublished |
| *Curvularia clavata* | BRIP:61680 | KU552205 | Khemmuk W, Shivas RG, Henry RJ, et al. (2016) Fungi associated with foliar diseases of wild and cultivated rice (*Oryza* spp.) in northern Queensland. Australasian Plant Pathology 45(3): 297–308. |
| *Curvularia eragrostidis* |  | no sequence | Bagwari A, Singh YP, Kumar J, Dhiman RC (2015) First report of *Curvularia eragrostidis* leaf spot on *Populus deltoides*. Forest Pathology 45(1): 86–87. |
| *Curvularia fallax* | HNHY001 | JQ360963 | Gao B D, Huang W, Xia H (2012) A new rice disease, black sheath spot, caused by *Curvularia fallax* in China. Plant Disease 96(8):1224. |
| *Curvularia fallax* | CBS 164.60 | MH857943 | Vu D, Groenewald M, De VM , et al. (2019) Large-scale generation and analysis of filamentous fungal DNA barcodes boosts coverage for kingdom fungi and reveals thresholds for fungal species and higher taxon delimitation. Studies in Mycology 92: 135–154. |
| *Curvularia inaequalis* | Wv3YBss2 | KC897663 | Amaradasa B, Amundsen K (2014) First report of *Curvularia inaequalis* and *Bipolaris spicifera* causing leaf blight of buffalograss in Nebraska. Plant Disease 98(2): 279. |
| *Curvularia inaequalis* | CBS 102.42 | MH856096 | Vu D, Groenewald M, De VM , et al. (2019) Large-scale generation and analysis of filamentous fungal DNA barcodes boosts coverage for kingdom fungi and reveals thresholds for fungal species and higher taxon delimitation. Studies in Mycology 92: 135–154. |
| *Curvularia lunata* | ITCC 8185.11 | no sequence | Avasthi S, Gautam AK, Bhadauria R (2015) Occurrence of leaf spot diseases on *Aloe vera* (L.) Burm.f. caused by *Curvularia* species from Madhya Pradesh, India. Biodiversitas Journal of Biological Diversity 16(1): 79–83. |
| *Curvularia lunata* | FBL02 | HG326308 | Akram W, Anjum T, Ahmad A, Moeen R (2014) First report of *Curvularia lunata* causing leaf spots on *Sorghum bicolor* from Pakistan. Plant Disease, 98(7): 1007. |
| *Curvularia lunata* | pingxiang | JQ701798 | Cui RQ , Sun XT (2012) First report of *Curvularia lunata* causing leaf spot on lotus in China. Plant Disease 96(7): 1068. |
| *Curvularia lunata* | QRF374 | KP278175 | Han C. Environmental influences on the expansion of germline tandem repeats in several species of Galapogas finches (unpublished) |
| *Curvularia lunata* | R1R | KP940576 | Majeed RA, Shahid AA, Ashfaq M, Saleem MZ, Haider MS (2015) First report of *Curvularia lunata* causing brown leaf spots of rice in Punjab, Pakistan. Plant Disease 100(1): 219. |
| *Curvularia lunata* | G712 | KT336201 | Liu T, Liu L, Hou J, Jing L (2016) First report of *Curvularia lunata* causing leaf spots on sweet sorghum (*Sorghum bicolor* (L.) Moench) in China. Plant Disease100(3): 652. |
| *Curvularia lunata* | taxon:5503 | LN879926 | Iftikhar S, Shahid AA, Nawaz K, Ali SW (2016) First report of *Curvularia lunata* causing fruit rot of tomato (*Lycopersicum esculentum*) in Pakistan. Plant Disease 100(5): 1013. |
| *Curvularia lunata* | WCCL | MG063428 | Xu G, Zheng F, Ma R, Zheng FQ, Zheng L, Ding XF, Xie CP (2018) First report of *Curvularia lunata* causing leaf spot of *Pennisetum hydridum* in China. Plant Disease 102(11): 2372. |
| *Curvularia lunata* | Cur | MG837719 | unpublished |
| *Curvularia lunata* | CBS 730.96 | MG722981 | unpublished |
| *Curvularia lunata* | L2858 | KC288113 | Santos DWCL, Padovan ACB, Analy SA, et al. (2013) Molecular Identification of Melanised Non-Sporulating Moulds: A useful tool for studying the epidemiology of Phaeohyphomycosis. Mycopathologia 175(5-6): 445–454. |
| *Curvularia malina* | FLS-2SS | JF812154 | Tomaso-Peterson M, Jo YK, Vines PL, et al. (2016) *Curvularia malina* sp. nov. incites a new disease of warm-season turfgrasses in the southeastern United States. Mycologia 108(5): 915–924. |
| *Curvularia malina* | WC11-1 | KY242610 | Zhang W, Liu J, Huo P, Huang Z (2017) *Curvularia malina* causes a foliar disease on *Hybrid bermuda* grass in China. European Journal of Plant Pathology 151(6): 557–562. |
| *Curvularia malina* | YBL-B8 | KY242611 |
| *Curvularia malucans* |  | no sequence | Pandey S, Kumar R, Mishra G, Giri K, Rishi R (2014) First report of *Curvularia malucans* causing severe leaf necrosis of *Curculigo orchoides* in india. Journal of Biology & Earth Sciences 4(2): B176-B178 |
| *Curvularia microspora* | HGUP 6272 | MF139088 | Liang Y, Ran SF, Bhat J, Hyde KD, Wang Y, Zhao DG (2018) *Curvularia microspora* sp. nov. associated with leaf diseases of *Hippeastrum* striatum in China. Mycokeys 29: 49–61. |
| *Curvularia microspora* | HGUP 6273 | MF139089 |
| *Curvularia microspora* | HGUP 6274 | MF139090 |
| *Curvularia microspora* | HGUP 6275 | MF139091 |
| *Curvularia microspora* | HGUP 6276 | MF139092 |
| *Curvularia microspora* | HGUP 6277 | MF139093 |
| *Curvularia microspora* | HGUP 6278 | MF139094 |
| *Curvularia microspora* | HGUP 6279 | MF139095 |
| *Curvularia microspora* | HGUP 6280 | MF139096 |
| *Curvularia nanningensis* | HGUP11000 | MH885316 | this study |
| *Curvularia nanningensis* | HGUP11001 | MH885317 |
| *Curvularia nanningensis* | HGUP11002 | MH885318 |
| *Curvularia nanningensis* | HGUP11003 | MH885319 |
| *Curvularia nanningensis* | HGUP11005 | MH885321 |
| *Curvularia prasadii* |  | no sequence | Singh N, Sharma P, Verma OP (2011) First report of *Achyrenthes aspera* leaf spot disease caused by *Curvularia prasadii* in India. New Disease Reports, 23(1): 32–33. |
| *Curvularia tuberculata* | R312 | KR704891 | Majeed RA, Shahid AA, Saleem MZ, Asif M, Zahid MA, Haider MS (2016) First report of *Curvularia tuberculata* causing brown leaf spot of rice in Punjab, Pakistan. Plant Disease 100(8): 1791. |
| *Curvularia tuberculata* | CBS 146.63 | MH858243 | Vu D, Groenewald M, De VM , et al. (2019) Large-scale generation and analysis of filamentous fungal DNA barcodes boosts coverage for kingdom fungi and reveals thresholds for fungal species and higher taxon delimitation. Studies in Mycology 92: 135–154. |
| *Curvularia verruculosa* | L7 | MF784436 | Hsiang T, Zheng L, Huang J (2010). First report of leaf spot caused by *Curvularia verruculosa* on *Cynodon* sp. in Hubei, China. Plant Pathology 54(2): 253. |
| *Curvularia verruculosa* | CPC 28792 | MF490825 | Marin-FelixY, Senwanna C, Cheewangkoon R, Crous PW (2017) New species and records of *Bipolaris* and *Curvularia* from Thailand. Mycosphere 8 (9): 1555–1573. |
